# Application of Fetal Membranes and Natural Materials for Wound and Tissue Repair

**DOI:** 10.3390/ijms252211893

**Published:** 2024-11-05

**Authors:** Marion Rouzaire, Loïc Blanchon, Vincent Sapin, Denis Gallot

**Affiliations:** 1 Obstetrics and Gynaecology Department, Centre Hospitalier Universitaire Clermont-Ferrand, 63000 Clermont-Ferrand, France; mrouzaire@chu-clermontferrand.fr; 2 “Translational Approach to Epithelial Injury and Repair” Team, Auvergne University, CNRS 6293, Inserm 1103, iGReD, 63000 Clermont-Ferrand, France; loic.blanchon@uca.fr (L.B.); vincent.sapin@uca.fr (V.S.); 3 Biochemistry and Molecular Genetic Department, Centre Hospitalier Universitaire Clermont-Ferrand, 63000 Clermont-Ferrand, France

**Keywords:** tissue repair, fetal membranes, natural materials, wound healing

## Abstract

The human fetal membrane is a globally accepted biological biomaterial for wound and tissue repair and regeneration in numerous fields, including dermatology, ophthalmology, and more recently orthopedics, maxillofacial and oral surgery, and nerve regeneration. Both cells and matrix components of amnion and chorion are beneficial, releasing a diverse range of growth factors, cytokines, peptides, and soluble extracellular matrix components. Beside fetal membranes, numerous natural materials have also been reported to promote wound healing. The biological properties of these materials may potentiate the pro-healing action of fetal membranes. Comparison of such materials with fetal membranes has been scant, and their combined use with fetal membranes has been underexplored. This review presents an up-to-date overview of (i) clinical applications of human fetal membranes in wound healing and tissue regeneration; (ii) studies comparing human fetal membranes with natural materials for promoting wound healing; and (iii) the literature on the combined use of fetal membranes and natural pro-healing materials.

## 1. Introduction

The three main phases of wound healing are inflammation, re-epithelialization, and tissue remodeling. Coordinated tissue mechanisms, including migration, proliferation, and adhesion, and involving cellular actors (inflammatory cells and fibroblasts) and proteins (extracellular matrix proteins, cytokines, and growth factors), enable the restoration of functional tissue after injury [1]. Aside from accidental burns and trauma, countless surgical wounds are generated yearly in standard medical treatments. Ensuring effective healing of both accidental and intentional injuries while reducing adverse esthetic effects on the patient and promoting the optimal restoration of tissue function is a key focus of clinical practice. Minor wounds in healthy individuals heal uneventfully, but more extensive injuries or the presence of various physiological conditions or common diseases, such as aging, infection, diabetes or other vascular issues, and cancer, can hinder the healing process [2].

Amniotic membrane (AM) is a globally accepted biological material for applications on skin, eyes, and in surgery to enhance wound healing and tissue regeneration [3,4]. Chorion has also shown promising results in oral and periodontal surgery [5]. Many useful biological properties have been reported (anti-inflammatory, antimicrobial, anti-fibrotic, anti-scarring, and epithelialization-promoting, among others) that make AM an ideal wound dressing. AM has been widely used as biological scaffold due to its 3D structural architecture and function and as a source of growth factors and cytokines. Both cells and the matrix of fetal membranes will release a diverse range of growth factors, cytokines, peptides, and soluble extracellular matrix components as been widely used as biological scaffold ted including act with maternal decidua [6,7].

Despite their known beneficial properties, fetal membranes have found few applications. AM is typically positioned over an injury and secured using sutures, adhesive, or extra bandages. The great fragility of the thin sheets and the need for sutures or adhesives to hold the membrane in place over the wound make them difficult to use. The complexity of handling and positioning these thin membrane sheets, along with the high costs associated with utilizing living cellularized tissue, have thus hampered the widespread adoption of AM wound healing products. Accordingly, new products derived from AM have been developed, offering easier production, storage, and application to wounds. These include a hydrogel delivery system for solubilized amniotic membrane [8,9].

Beside fetal membranes, numerous natural materials have been reported to possess wound healing ability [10,11]. The biological properties of these materials may potentiate the pro-healing action of fetal membranes, and so their combined use could have applications in medicine. These natural materials could also be better candidates than fetal membranes for developing wound scaffolds if their pro-healing properties are found to be superior to those of fetal membranes. They also could be easier to use in clinical practice, and the rare risk of transmitting infections from donors could be avoided by using natural materials compared to fetal membranes.

This review gives an up-to-date overview of (i) clinical applications of human fetal membranes in wound healing and tissue regeneration; (ii) studies comparing human fetal membranes and natural materials for promoting wound healing; and (iii) the literature on the combined use of fetal membranes and natural pro-healing materials.

## 2. Role, Structure, and Biological Properties of Human Fetal Membranes

### 2.1. Role

The fetal membranes are complex, transient tissues that enclose the fetus throughout pregnancy, acting as a barrier between the fetal and maternal compartments and providing mechanical protection against external shocks. Their strength and shock-absorbing capacity are due to possessing collagen content and multi-layer structure (natural cross-linking). In addition to physical protection, fetal membranes provide an immunological barrier, ensuring biological protection against ascending vaginal flora bacteria. Numerous studies have highlighted the anti-inflammatory and antimicrobial roles of fetal membranes [12,13,14] that make them particularly attractive for use in tissue engineering and clinical applications [4,6]. Thanks to this immune role, the fetus and the membranes themselves are protected from external infectious agents present in the reproductive tract. Being in direct contact with amniotic fluid, fetal membranes also play an essential role in the regulation of amniotic fluid homeostasis (volume, composition, and renewal). They are involved in the initiation of parturition through their rupture at the end of pregnancy.

### 2.2. Structure

The fetal membranes comprise two joined histologic layers. The innermost layer, the amnion, lies in direct contact with the amniotic fluid in which the fetus bathes. The outermost layer, the chorion, is attached to the underlying maternal decidua. The first studies describing the anatomical and histological structure of the fetal membranes were conducted by Golden Bourne in the 1960s [15,16,17]. Much further research in subsequent years culminated in their current very complete and detailed description [18].

#### 2.2.1. Amnion

**Figure 1:**
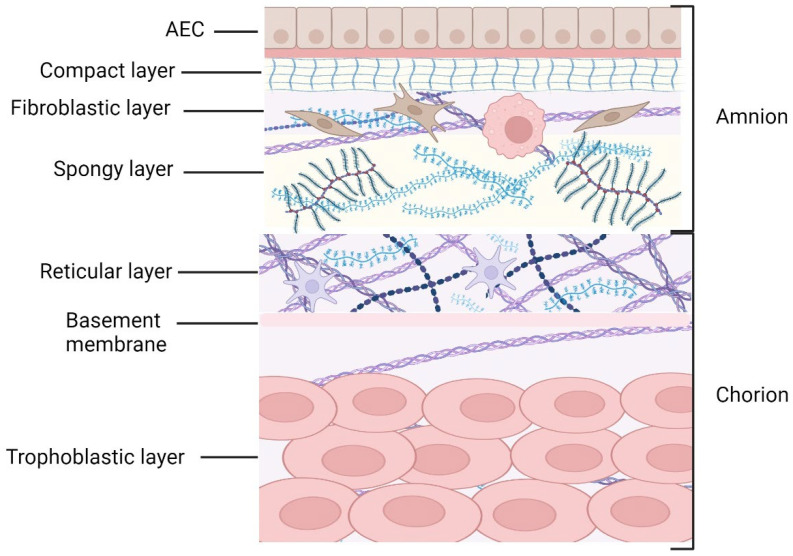
Structure of human fetal membranes. AEC: amniotic epithelial cells. Created in BioRender.

The amnion is neither vascularized nor innervated. It comprises five fine layers with a total thickness of approximately 0.1 mm (1). The first is a monolayer of cuboidal amniotic epithelial cells (AECs), which line the amniotic cavity. This epithelium is supported by a basement membrane composed of type III, IV, and V collagens and non-collagenous glycoproteins (such as laminin and fibronectin) secreted by the epithelial cells. The third layer, known as the compact layer, forms a real fibrous skeleton that strengthens the amniotic layer and thus helps keep the fetal membranes intact [18]. It is made up of a dense network of fibrillar collagens (mainly type I and III) organized in bundles, which are themselves secreted by the underlying fibroblastic layer. The fibroblastic layer is the thinnest layer of the amnion, consisting of amniotic mesenchymal cells (AMCs), also named fibroblasts, and rare macrophages embedded in an extracellular matrix. The last layer of the amnion, the spongy layer, makes contact with the chorion. It is rich in hydrated proteoglycans and absorbs the many deformations caused by both fetal movements and external shocks. It also forms a sliding zone between the amnion and the chorion (Figure 1).

The organization of the collagen fibers is essential to ensuring the amnion’s resistance to external pressure. In addition to the dense fibrous network of the compact layer, the strength of the amnion is ensured by collagen bridges linking the basement membrane to the deeper layers of the amniotic membrane. These fibers, composed of type V and VI collagens, are visible under a microscope as ribbons organized in parallel trabeculae [19]. In addition, large amounts of hyaluronan are found in the amnion, which facilitates cell movement [20].

#### 2.2.2. Chorion

The chorion is much thicker than the amnion (up to 0.4 mm thick) but much less resistant to tensile forces. It is composed of three layers. The first, in contact with the amnion, is called the reticular layer. It is made up of a fibrous network rich in collagen and proteoglycans (especially decorin) in which spindle and stellate cells are embedded. This structure adheres firmly to the underlying chorionic basement membrane. The latter, rich in type IV collagen, laminin, and fibronectin, supports the trophoblastic layer through anchoring and connecting fibers in the trophoblast. The trophoblastic layer is formed by a stack of 2–10 layers of trophoblastic cells. It is the outermost structure of the fetal membranes and makes close contact with the cells of the maternal decidua.

### 2.3. Biological Properties

**Figure 2:**
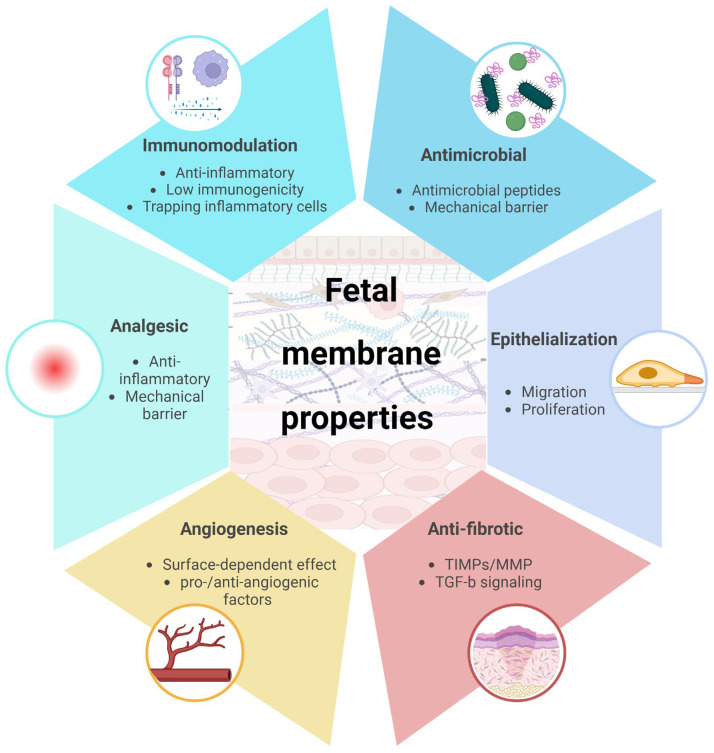
Pro-healing properties of human fetal membranes. Created in BioRender. Blanchon, L. (2024) BioRender.com/o54w144.

Amnion and chorion are of special interest for clinical applications because of their anti-inflammatory and immunosuppressive properties. Other biological properties such as antimicrobial, promoting epithelialization, anti-fibrotic action, analgesic effects, and proangiogenic and antiangiogenic activities are widely described in the literature [6,7]. Both cells and matrix components of the membranes are beneficial by releasing a diverse range of growth factors, cytokines, peptides, and soluble extracellular matrix components that regulate several mechanisms of the healing cascade [4,21,22,23] (Figure 2).

#### 2.3.1. Immunomodulation

In the literature, anti-inflammatory properties of fetal membranes are mainly explained by the secretion of anti-inflammatory signals such as IL10, IL4, HGF, and PGE2 and the suppression of pro-inflammatory cytokines TNFα, IL1, IL6, and IL8 [7,24,25]. AM prevents the proliferation of T and B cells, reduces the inflammatory characteristics of monocytes, macrophages, dendritic cells, neutrophils, and natural killer cells, and promotes the development of regulatory T cells and anti-inflammatory M2 macrophages [26]. Another anti-inflammatory mechanism highlighted on the ocular surface is the ability of AM to attract and trap infiltrating inflammatory cells [27]. Moreover, the amniotic membrane exhibits low immunogenicity due to the expression of HLA-G and Fas ligands and the absence of MHC Class I surface markers [28,29]. Their capacity to regulate and inhibit immune responses makes them invaluable to treat chronic inflammatory diseases and to prevent rejection in transplantation.

#### 2.3.2. Antimicrobial Effect

Amniotic cells have antibacterial properties that can be enhanced by inflammatory signal inducers [30]. They secrete antibacterial peptides including elafin, HBD-2, HBD-3, and cathelicidic LL-37 [31,32]. Moreover, when used on a wound, AM adheres to the edges of the injury and constitutes a mechanical barrier against potential external bacteria.

#### 2.3.3. Re-Epithelialization

Fetal membranes are able to support cell adhesion because of their structural proteins, collagen types I, VI, and VII, laminin, fibronectin, and vitronectin. Several signaling pathways have been described to explain the pro-migration and proliferation effects of FM, including modulation of JNK and MEK MAP kinase signaling pathways to promote focal adhesions [33] and modulation of the TGF-β signaling pathway [34,35]. AM-induced migration seems to involve the recruitment of the EGF receptor, MEK1 activation, and c-Jun phosphorylation. Additionally, AM partially suppresses TGF-ß-stimulated nuclear localization of R-Smads in HaCaT and Mv1Lu cells. Among the various cytokines that impact wound healing, TGF-ß plays a pivotal role in regulating numerous cellular responses throughout all three phases of wound healing. Its signaling is cell-type-specific and tightly regulated. It was proposed that AM may secrete a cytokine cocktail that, by modulating TGF-ß signaling in keratinocytes and stimulating c-Jun phosphorylation, initiates and promotes the re-epithelialization process of wounds [34,35,36,37].

#### 2.3.4. Anti-Fibrotic Action

Fibrosis appears in many chronic inflammatory diseases and is characterized by the abnormal deposition of extracellular matrix components, which disrupt normal tissue structure and function. Molecular mechanism involves activation of the coagulation pathway, followed by acute inflammation and the activation of innate immune mediators. These inflammatory and immune mediators lead to the transformation of inactive fibroblasts into myofibroblasts, which produce additional ECM and exert tractional forces on the ECM, distorting tissue architecture and ultimately causing fibrosis.

The amniotic membrane can act on fibrosis at various stages, resulting in reduced scarring and the preservation of tissue structure and function. First, it was demonstrated that amniotic membrane stroma can maintain keratocytes in cultures and prevent them from differentiating into myofibroblasts [38]. Moreover, myofibroblasts seeded onto a cryopreserved amniotic membrane stromal surface reverted back to the fibroblast phenotype. The authors concluded that amniotic membrane stroma contains soluble factors that can regulate mesenchymal cell differentiation [39]. Additionally, hAM can selectively counteract the effects of TGF-β, which is involved in overexpression of ECM components, leading to fibrosis during tissue repair.

#### 2.3.5. Angiogenesis

During angiogenesis, endothelial cells move from existing blood vessels into nearby tissues, where they proliferate and form new cell-to-cell connections and tubular structures of new capillaries. Both amniotic epithelial and mesenchymal cells exhibit proangiogenic potential in vitro. Amniotic epithelial cells secrete proangiogenic cytokines, including angiogenin, EGF, IL-6, and monocyte chemoattractant protein (MCP)-1, contributing to myocardial tissue regeneration in a myocardial infarction rat model [40]. Amniotic mesenchymal cells increase blood flow and capillary density in hindlimb ischemia of mice [41,42] and secrete VEGF-A, angiopoietin-1, and FGF-2, IGF-1, EGF, IL-8, and ki-67 [22]. In contrast, amniotic membranes also have anti-angiogenic effects demonstrated on the eyes [43,44]. Molecular mechanisms involve secretion of collagen IV, laminin, integrins 4/6, endostatin, thrombospondin-1, and TIMP-1/2/3/4 [22]. A study has highlighted a dual effect of the amniotic membrane on angiogenesis, dependent on the surface side. In this study, a layer of dorsal skin of rats was removed, and AM was implanted in either an epithelial side-up or mesenchymal side-up position. The epithelial side of the AM had an anti-angiogenic effect, whereas the mesenchymal side promoted angiogenesis [45].

#### 2.3.6. Analgesic Effect

The analgesic effect of fetal membranes has been highlighted in surgery and burn treatment. The effect of fetal membranes on pain may be due to their immunomodulatory effect. Anti-inflammatory action of fetal membranes contributes to reducing clinical symptoms, including pain [6]. The use of AM wrapping to envelop the injury site at the end of surgical procedure (tendon and nerve repair) may also contribute to limiting pain by protecting the wound edges from external pathogens. Clinical application of fetal membranes to promote wound healing.

## 3. Clinical Application of Fetal Membranes to Promote Wound Healing

The clinical use of fetal membranes began with skin transplantation in 1910 [46]. It was followed by other applications in dermatology to heal burned and ulcerated skin and in ophthalmology to treat conjunctival defects in 1940 [47]. Fetal membranes now serve a wide range of other medical specialties, including applications in orthopedics, nerve regeneration, maxillofacial and oral surgery, chronic venous ulcers, and lung, gynecological, obstetrical, and fibrotic pathologies.

**Figure 3:**
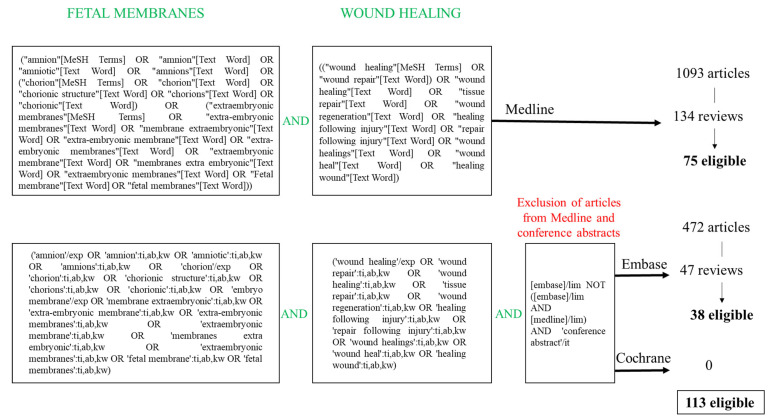
Selection of the reviews on fetal membrane applications for wound healing. In green, keywords used to build the equation; in red, explanation that articles from Medline and conference abstracts were excluded from Embase and Cochrane requests; in bold, number of articles eligible.

We searched the PubMed, Embase, and Cochrane databases for applications of fetal membranes to wound healing using the keywords “fetal membranes” and “wound healing”. Extractions were made on 7 June 2024. We excluded reviews concerning wound healing of fetal membranes in cases of premature rupture of membrane or injury, applications of AM-derived mesenchymal stem cells, amniotic fluid-derived stem cells, secretome, exosome, pre-eclampsia, and equine wound management. Eligible publications were selected on the basis of their title and abstract. We collected a list of 113 reviews relating to clinical applications of fetal membranes for wound healing and tissue repair (Figure 3).

**Figure 4:**
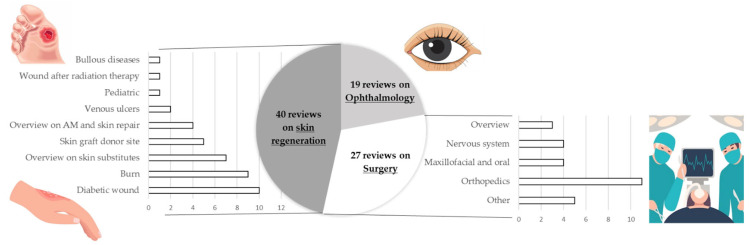
Reviews on applications of fetal membranes for wound healing.

Of the 113 eligible reviews, 25 gave overviews of clinical applications, how fetal membranes were used, and the molecular mechanisms involved in the healing process. Two reviews explored fetal membranes in combination with other natural materials for wound healing applications (honey and hyaluronic acid). The remaining 86 reviews were specific to pathologies in their application fields (Figure 4).

Clinical use of fetal membranes in wound healing and tissue repair was queried in the ClinicalTrials.gov database. We obtained a list of 144 trials after excluding those evaluating amniotic fluid derivatives (Zofin, Dermacyte^®^ liquid) and amnion-derived multipotent cell secretomes (ST266) (Supplementary File S1).

Amnion is commonly used for clinical applications; chorion has been used less often [48,49]. Chorion has been used mostly in oral and periodontal surgery, with results equal to or better than amnion [5]. Recently, hypothermic storage of chorion was found to preserve its structural characteristics and its functional capacity as a scaffold to support tissue growth [50]. Numerous fetal membrane-based treatments for wound healing and tissue repair are available in the clinic, including EpiFix^®^, Grafix^®^, Life Patch^®^, AmnioExcel^®^, AmnioBand^®^, HSAM^®^, NEOX^®^1K, AMNIODERM+^®^, NuCel^®^, AmnioFIX^®^, ProKera Plus^®^, Visio-AMTRIX^®^, Orion TM^®^, Clarix^®^ 1k, Omnigen^®^, PalinGen Flow^®^, Visio-AMTRIX^®^, AMEED^®^, and BioXclude^®^.

### 3.1. Skin Repair

Fetal membranes have been widely used on skin to treat both acute and chronic wounds, including skin graft donor sites [51,52,53,54,55], diabetic foot ulcers (DFUs) [56,57,58,59,60,61,62,63,64], venous leg ulcers [65,66], neuropathic ulcers, pressure sores, and burns [67,68,69,70,71,72]. Wound management includes preventing infections, promoting tissue regeneration, achieving wound closure, and overseeing scar formation and remodeling. Various wound-care products are on the market, such as basic protective layers, hydrogels, dressings infused with pro-healing substances, and synthetic skin substitutes, all designed to aid in surface closure [73]. Various commercial AM-derived scaffolds are produced in the USA and are undergoing clinical trials for application in dermatology. Meta-analyses found on the use of fetal membranes to repair skin wounds are described below.

#### 3.1.1. Burn Wounds

The role of amniotic dressings in burn wounds has been evaluated by two recent meta-analyses. Chao et al. included 11 randomized controlled trials (RCTs) with 816 patients. They found that fetal membrane was more effective than conventional methods, silver sulfadiazine, and polyurethane membrane [71]. Notably, treatment with fetal membrane was less effective than honey dressing. The second study was published in Chinese. Results reported in the abstract also indicated efficacy in favor of new pro-healing materials compared to fetal membranes, especially *Aloe vera* gel, which gave a shorter wound healing time than amnion and honey dressing, which presented the lowest infection rate [74].

#### 3.1.2. Skin Graft Donor Site

Use of AM to heal a skin graft donor site in split-thickness skin grafting was studied in one meta-analysis [53]. This reconstructive technique, used to enhance healing of burns, chronic ulcers, and tissue loss resulting from surgery or trauma, generates a secondary surgical site named the skin graft donor site. Various dressings can be used to enhance the healing of donor sites. The ideal dressing should facilitate healing, minimize pain, decrease infection risks, prevent hypertrophic scar formation, be easy to apply, and be cost-effective. Liang et al. included seven studies with 219 patients. They found a benefit in healing time (mean difference −3.87 days) and healing rate (RR = 1.61) with AM compared to the current standard of care (SOC) [53]. The benefit of biological dressings versus non-biological dressings was confirmed in a meta-analysis including eight RCTs [52]. Neither study found any difference in infection rate or pain alleviation.

#### 3.1.3. Diabetic Wounds

Chronic wounds in diabetes can result in impaired skin regeneration owing to high oxidative stress, poor angiogenesis, insufficient collagen hyperplasia, and accumulation of M1 macrophages correlated with prolonged expression of pro-inflammatory cytokines. Three meta-analyses were found for treatment of diabetic foot ulcers (DFUs) using fetal membranes. SOC was pressure relief, debridement, infection control, and revascularization when necessary [75]. In some cases, SOC may not be sufficient, prompting new approaches such as fetal membranes. The safety profile of fetal membranes and their benefit for promoting healing as an adjuvant to SOC was found in two recent meta-analyses [56,57]. The third meta-analysis included all RCTs on new medical dressings and ranked the best healing strategies for DFU. Amniotic membrane came in the top three in terms of healing rate, with platelet-rich plasma and epidermal growth factor. We note that the use of honey dressing has also been studied in this context and presented a better healing time than traditional and alginate dressings [76].

### 3.2. Ophthalmology

The amniotic membrane is of particular interest in ophthalmology for its ability to limit inflammation, promote re-epithelialization, and reduce pain. It has been used in more than 20 ophthalmic procedures to treat persistent epithelial defects, corneal perforations, limbal stem cell deficiency, bullous keratopathy, conjunctival reconstruction after neoplasia excision, pterygia, fornix reformation, and acute ocular burns [77]. Amniotic stroma has the ability to merge with the host corneal tissue through the development of adhesion structures (hemidesmosomes and desmosomes), which stabilize the regenerating corneal epithelium [78]. Commercial frozen and dried preparations of AM are available for ophthalmologic surgery. However, the level of evidence supporting its use is limited, and RCTs are few in number. One RCT was conducted to evaluate AM in pterygium surgery, with a result in favor of conventional treatment [79]. One RCT compared AM transplantation with conventional management (tarsorrhaphy and bandage contact lenses) in eyes with refractory neurotrophic corneal ulcers without finding any difference between groups [80]. The only benefit of AM transplantation was found on moderate acute ocular burns in terms of pain reduction and promotion of early epithelialization. This RCT was conducted on 20 eyes treated by AM transplantation versus 24 control eyes treated with a conventional medical approach [81].

Human AM is commonly used as a vehicle for delivering cultured limbal stem cells to the cornea. Additionally, it serves as a cell-free dressing owing to its anti-angiogenic and anti-inflammatory properties. The biodegradation time of the AM varies depending on its processing and the specific storage conditions in tissue banks. Biodegradation is also influenced by individual conditions of the eye to which the amniotic membrane is transplanted. Despite rigorous screening of maternal donors prior to membrane use, there remains a residual risk of viral disease transmission that cannot be entirely eradicated [82].

Novel strategies of cell therapy and tissue engineering are currently being developed to promote corneal epithelial regeneration after injury [83]. Many stem cells and scaffolds have been tested, including decellularized amnion extract (dAE) to support retention of stem cells on damaged ocular surfaces, thus helping corneal epithelial regeneration.

Among the various forms of administration tested, dAE in situ gel (with temperature-dependent Poloxamer 407 as the matrix) was the most efficient in reducing healing time for epithelium and hastening recovery for stromal opacity and thickness [84]. The biological mechanism seems to involve an overexpression of leucine-rich and immunoglobulin-like domain protein 1 (LRIG1), which is involved in self-renewal of corneal epithelium after injury and corneal transparency [85].

Bioactive factors released by amniotic extracts are present only for a short time after the transplant and gradually disappear. New scaffolds are currently being developed to improve the efficacy of AM in promoting wound healing.

### 3.3. Orthopedics

Applications of AM in orthopedics are currently being developed [86]. In a clinical setting, AM has been evaluated for the treatment of plantar fasciitis in two pilot studies, against placebo [87] or corticosteroid injection [88]. A benefit was found compared to placebo, but equivalent results were found when AM was compared to traditional corticosteroid injection.

Adding an AM injection to tennis elbow surgery with Tenex™ was recently assessed in one clinical retrospective study but did not present any benefit [89]. AM has also been evaluated for the treatment of knee osteoarthritis. Feasibility of a single intra-articular injection of amniotic suspension allografts was established [90]. Its efficacy is currently being tested in clinical trials (NCT06000410, NCT05796765, NCT04636229, NCT03485157, NCT03337243).

Freeze-dried AM was tested to promote healing and prevent adhesion after flexor tendon surgery. It was used to wrap broken ends of the tendon. In one study conducted by Liu et al. on 89 patients, the authors concluded that AM was a safe, effective, and completely absorbable barrier material that could reduce tendon adhesion, the most common complication reported after tendon surgery [91]. By contrast, a Finnish team planning to conduct a pilot study on 10 patients terminated it after five inclusions owing to unfavorable results. One patient had extensive stiffness and was subject to tenolysis and joint release associated with focal fibrosis. Another patient had a repair failure. The other three patients showed fair-to-good results [92].

### 3.4. Other Fields of Surgery

Several surgical specialties have used AM for its pro-healing properties, biocompatibility, and safety [4,93].

#### 3.4.1. Nerve Repair

Surgical applications to reconstruct peripheral nerves after injury are still being developed. Optimal nerve repair should restore both motor and sensory functions. Two different strategies are implemented to treat proximal nerve injuries. The gold standard includes direct nerve restoration and autograft. The second involves the use of engineering tissue tubes and was demonstrated to be as effective as nerve grafting for restoring separations of more than 4 cm [94].

Several studies evaluated applications of AM in nerve repair, but its clinical use is not widespread. The use of AM wrapping to envelop the injury site at the end of the surgical procedure decreases the formation of scar tissue, reduces adhesion, and improves functional recovery. Moreover, human AM protects the endoneural site from inflammation [95,96]. A tubulization technique combining amnion and muscle graft was tested on five patients with median nerve damage at the wrist with a gap of >4 cm [97]. Nerve restoration was demonstrated after one week, and myelinated fibers appeared after three weeks.

#### 3.4.2. Maxillofacial and Oral Surgery

Several studies highlight the utility of fetal membranes for periodontal tissue repair. Applications were evaluated in cleft palate and tumor reconstruction, peri-implant surgery, gingival recession treatment, intrabony and furcation defect treatment, alveolar ridge preservation, maxillary sinus membrane repair, and large bone defect reconstruction [5,98,99,100,101]. Both amniotic and chorionic membranes were used.

Two randomized trials were run to evaluate the use of AM in guided tissue regeneration for surgical treatment of interdental defects. One trial on 30 patients demonstrated a benefit of using AM combined with bone graft compared to bone graft alone [102]. A strong anti-inflammatory effect of AM was found, with an improvement in periodontal variables. The second study conducted on 10 patients compared AM and collagen membranes and showed equivalent effects of the two membranes on periodontal variables. AM had the advantage of not inducing gingival recession, whereas an increase in gingival recession was shown with collagen membrane [103].

The use of chorion membrane and amniochorionic membrane to treat periodontal intrabony defects was compared to controls in four randomized trials. One RCT on 22 patients found no difference between amniochorionic membrane and demineralized bone matrix (control) at 6 months for all clinical variables [104]. The team of Kothiwale et al. compared chorionic membrane to open flap debridement without membranes (control) in three studies conducted on 5, 10, and 10 patients, respectively. They found a benefit of using chorionic membrane for all investigated variables [105,106,107].

The benefit of using chorionic membrane for furcation defect treatment was shown in one RCT on 14 patients [108]. Chorion was also found to improve treatment of gingival recessions in four RCTs. Compared to amnion, chorionic membrane showed either equal or better results in this field [109,110,111].

One RCT on 10 patients tested the use of AM placed over the surgical wound during peri-implant surgery. No difference was found in the final outcome, but AM seemed to shorten healing time and reduce pain [112].

The use of fetal membranes, especially chorionic membranes, has yielded promising results in pilot studies conducted in maxillofacial and oral surgery. Further RCTs comparing fetal membranes and standard surgery are needed to seek clinical evidence demonstrating their advantages.

#### 3.4.3. Gynecology

In gynecology, several clinical trials have demonstrated a benefit in using both fresh and dried AM to prevent intrauterine adhesions (IUAs) after surgical procedures [4].

Amnion has also been used in reconstructive vaginoplasty surgery with promising results [113,114], but no RCT has been conducted. Cryopreserved AM has also been used as a graft to cover vaginal defects after partial excision of the mesh erosion in eight patients with lesions 5–25 mm in size. No intraoperative complications were found. Follow-up at 27 months found only one patient with recurrent erosion. The authors highlighted AM graft as an economic alternative method to treat complex vaginal mesh erosions [115].

#### 3.4.4. Other

Applications of AM were recently reported in clinical case studies on other surgical areas, such as for the treatment of cryptoglandular anal fistulas in 2022 [116] and to cover pancreatic anastomosis after pancreaticoduodenectomy in 2019 [117].

One RCT on 35 patients found a benefit of using AM transplantation in carpal tunnel syndrome release surgery [118]. Outcomes were assessed using three different questionnaire scores (Boston Carpal Tunnel Syndrome Questionnaire, Disabilities of the Arm and Shoulder, and Hand, and Historical–Objective Scale) at baseline and 15 days, 1, 3, 6, and 12 months after surgery. One RCT on 60 patients found a benefit of applying AM after tonsillectomy. Pain was reduced in the AM group, but no significant difference was found for bleeding [119].

## 4. Fetal Membranes and Natural Materials to Promote Healing

Naturally occurring materials have been used for millennia in wound healing practices. Such materials are abundant in various plants and animals, making them a readily available resource for wound treatment. Their efficacy in promoting healing has been demonstrated in traditional Chinese and Indian medicine. Traditional therapies based on natural materials are often advocated and may be more affordable and accessible than synthetic alternatives. Natural materials are still valued in wound care for their healing properties. Several have been used in nanoformulations to promote wound healing [10].

We drew up a list of natural materials exhibiting bioactive properties useful for wound healing based on literature reviews on the subject [10,11] (Table 1).

**Table 1:** List of articles on natural materials and fetal membranes to promote healing.

Material (Search Term)	All *	Excluded Articles	Included Articles	Combined Use with Fetal Membranes	Comparison with Fetal Membranes
Chitosan	18	5	13 (Table 2)	6	7
Hyaluronic acid	51	43	8 (Table 3)	5	3
PLAT platelet-derived growth factor	36	34	2 (Table 4)	1	1
Honey	4	1	3 (Table 5)	1	2
Epigallocatechin-3-gallate (EGCG)	3	1	2	1 (used for AM preparation)	1
Lysine	37	36	1	1 (used for AM preparation)	0
*Aloe vera*	1	0	1	1	0
Dichloromethane	2	2	0		
Hexane	6	6	0		
Shikonin	1	1	0		
Lectin	63	63	0		
Carbohydrates	22	22	0		
Bilirubin	15	15	0		
Minerals	15	15	0		
Soybean	12	12	0		
Wheatgerm	11	11	0		
Oleic acid	6	6	0		
Resveratrol	5	5	0		
Carotenes	4	4	0		
Vitamin B	4	4	0		
Amino acids	3	3	0		
Beta-carotene	3	3	0		
Metformin	3	3	0		
Propolis	3	3	0		
Quercetin	3	3	0		
Alpha-chymotrypsin	2	2	0		
Astragali radix	2	2	0		
Caffeic acid	2	2	0		
Essential oil	2	2	0		
Thymol	2	2	0		
Anthocyanin	1	1	0		
Apigenin	1	1	0		
Apigenin-7-*O*-glucoside	1	1	0		
Baicalin	1	1	0		
Bromelain	1	1	0		
Carvacrol	1	1	0		
*Catharanthus roseus*	1	1	0		
Danggui buxue	1	1	0		
Eucalyptus	1	1	0		
Fenugreek	1	1	0		
Hexanoic acid	1	1	0		
Kaempferol	1	1	0		
Luteolin	1	1	0		
Oleanolic Acid	1	1	0		
Polyphenols	1	1	0		

* number of articles found on fetal membranes and respective material.

Pubmed equations used to search articles on fetal membranes associated with or compared to natural materials and pro-healing materials investigated were provided in Supplementary File S1.

### 4.1. Chitosan

Chitin is one of the most prevalent polysaccharides after cellulose. Deacetylation of chitin yields a polysaccharide soluble in aqueous acidic media called chitosan. It is the most important derivative of chitin and can be extracted from the exoskeleton of marine crustaceans (shrimp and crabs), insects, fungi, and yeasts. Its structure is a copolymer of glucosamine and *N*-acetylglucosamine units connected by 1–4 glucosidic linkages. It can be used to create hydrogels, films, fibers, or sponges [120]. It is biodegradable, non-toxic, hydrophilic with an affinity for proteins, and it possesses bacteriostatic and hemostatic properties. Chitosan finds various applications in agriculture, waste treatment, food, cosmetics, biology, and pharmaceuticals. In biopharmaceuticals, it is used as a drug delivery system to enhance drug absorption. It has also been used for gene transfection and as a scaffold for tissue repair. Its pro-healing properties seemed to involve infiltration of polymorphonuclear cells followed by production of collagen [121] and promotion of fibroblast and keratinocyte proliferation. This effect was highly dependent on the degree of deacetylation, with high degrees of deacetylation showing more activity [122]. The authors of this last study suggested that chitosan interacts with growth factors present in the serum, potentiating their effect, but it was not clearly demonstrated. This point supports the fact that the combination of fetal membranes and chitosan could act molecularly in synergy. In addition, chitosan exhibits antibacterial properties due to its ability to interact with negatively charged molecules present in bacterial membranes [123]. In addition, the use of chitosan to deliver AM extract could be a promising approach for ocular reconstruction to prolong the viability of bioactive factors provided by AM and increase biological activity.

Literature comparing chitosan and fetal membranes in tissue repair is listed in Table 2.

**Table 2:** Amniotic membranes and chitosan as promising strategies for tissue repair.

Reference	Material		Intended Clinical Application	Type of Study	Model	Evaluation Time	Biological Properties
A—Comparison of Chitosan and Amniotic Membranes							
	AM	Chitosan					
Li et al., 2020 [124]	Fresh AM	IU injection	Prevention of IUAs	RCT	100 patients	3 months	AM: prevented adhesions (benefit compared to chitosan) and increased endometrial thickness
Yeh L.K. et al., 2009 [125]	AM	Chitosan membrane	OS reconstruction	in vitro	Bovine corneal epithelial cells	7 days	CM and AM: preserved phenotype, growth of corneal epithelial cells with minimal toxicity
Feng Y. et al., 2014 [126]; Zhu et al., 2006 [127]		Gelatin–chitosan (GC)	OS reconstruction	in vitro	Rabbit conjunctival epithelial cells	14 days	GC: proliferation of conjunctival fibroblasts and growth of the explants
Kamarul T. et al., 2014 [128]	AM	PVA/NOCC scaffold	Cutaneous wound	in vivo (animal model)	Rats –subcutaneous implementation (n = not available)	15 days	AM and PVA: low signs of toxicity
Dong R. et al., 2020 [129]	PCL-amnion nanofibrous membrane	Medical chitosan hydrogel	Nerve wrapping	in vivo (animal model)	Rats—sciatic nerve compression model (*n* = 90)	12 weeks	AM: decreased adhesion and inflammation of nerve tissue Increased proliferation of Schwann cells, nerve growth factors
Dong R. et al., 2022 [130]	PCL-amnion nanofibrous membrane	Medical chitosan hydrogel	Nerve wrapping	in vivo (animal model)	Rats—sciatic nerve compression model (*n* = 96)	12 weeks	AM: decreased peripheral nerve adhesion of pro-inflammatory M1 macrophages, type I and III collagen Increased recovery of nerve conduction; Schwann cells, nerve growth factor
Washburn S. et al., 2010 [131]	AM coated with halofuginone on both sides	AM coated with halofuginone and chitosan	Preventing peritoneal adhesions (wrapping)	in vivo (animal model)	Rats with uterine horn injury (*n* = 60)	2 weeks	Equal: decreased % of animals with adhesions, % of animals with moderate and severe adhesions
B—Combined use of Chitosan and Amniotic Membranes							
Dadkhah Tehrani F. et al., 2022 [132]	H_2_O_2_-loaded PLA microparticles chitosan hydrogel covered with a layer of dAM		Wound dressing with O2-generating capacity	in vitro	3T3 cells	Up to 7 days	Stable, oxygen release for at least 7 days, supported cellular growth, adhesion, and morphology
Rana M.M. et al., 2020 [133]	dAM gel in association with a covering membrane composed of collagen and chitosan		Cutaneous burn	in vivo (animal model)	Rats—burn model (*n* = not available)	19 days	Improved wound healing, re-epithelialization, and closure by wound contraction
Shabani A. et al., 2020 [134]	AM extract-loaded nanoparticles (dextran sulfate chitosan)		OS reconstruction	in vitro	endothelial cells	10 days	Longer and significantly increased biological activity in vitro (anti-angiogenic effect)
Momeni M. et al., 2018 [135]	AM extract gel based on chitosan/PVP gel containing AM extract		Cutaneous burn	in vivo (animal model)	Rats—burn model (*n* = 42)	_	Increased epidermal and dermal regeneration, formation of granulation tissue, fibroblast proliferation, blood capillary formation, developing collagen bundles
Bakhshandeh H. et al., 2021 [136]	AM extract nano-encapsulated in chitosan–dextran nanoparticles, decorated on artificial cornea		Corneal transplantation (artificial cornea)	in vitro	HUVE cells	Up to 5 days	Release of anti-angiogenic factors: thrombospondin-1, endostatin, and heparin sulfate proteoglycan
Bankoti K. et al., 2020 [137]	Carbon nanodot decorated with dAM extract, chitosan hydrogel, and associated with hAMSCs		Cutaneous wounds	in vitro	Scratch assay	21 days	Promoted angiogenesis, collagen deposition, reepithelialization, and formation of organized dermal epidermal junctions

IUA: intrauterine adhesion; dAM: decellularized amniotic membrane; PCL: polycaprolactone; PLA: polylactic acid; PVA/NOCC: poly(vinyl alcohol)/N, O-carboxymethyl chitosan; H_2_O_2_: hydrogen peroxide; HUVE cells: human umbilical vein endothelial cells; 3T3 cells: mouse embryonic fibroblasts.

The one clinical trial comparing AM and chitosan was in gynecology, conducted with the aim of preventing recurrence of intrauterine adhesions (IUAs) in women with moderate to severe IUAs after transcervical resection of adhesion.Benefit was found for AM compared to chitosan in recurrence rate of adhesions in the first month (chitosan: 47.9%, AM: 15.4%) and 3 months after surgery (chitosan: 37.5%, AM: 3.8%). An increase in endometrial thickness was found in the AM group [124].

Biocompatibility and toxicity of amnion and chitosan were compared in a rat model. Five days after subcutaneous implementation, chitosan-implanted rats exhibited inflammatory infiltration, while no such low toxic tissue response was observed in amnion-implanted rats [128].

In ophthalmology, AM transplantation is a highly effective approach for enhancing corneal wound healing while preserving phenotypes of corneal epithelial cells. Exploring alternative synthetic and biocompatible substitutes for treating ocular surface disorders remains relevant. The chitosan membrane has been tested in vitro and was demonstrated to preserve the phenotype of bovine corneal epithelial cells as effectively as AM [125]. Gelatin–chitosan membranes have also been tested in vitro for ocular surface reconstruction and showed promising results [127], but no in vivo study or clinical data have yet been published. Amniotic membranes remain the gold standard scaffold for transplantation of limbal epithelial stem cells for ocular surface reconstruction [126,138].

Two studies were conducted in a rat model of sciatic nerve compression to compare the efficacy of polycaprolactone (PCL)-amnion nanofibrous membrane and chitosan hydrogel for preventing adhesion formation. Amnion was coated with PCL to prolong its maintenance in vivo. In 2020, Dong et al. compared 30 rats in a PCL-amnion group, where amnion was wrapped and sutured, versus 30 rats in a chitosan group (injected) and 30 controls. The use of PCL-amnion significantly reduced nerve tissue adhesion and inflammation during the recovery phase [129]. In 2022, the same authors conducted a new study on 96 rats and confirmed that PCL-amnion significantly reduced peripheral nerve adhesion and promoted nerve regeneration while decreasing intraneural macrophage invasion [130]. Moreover, the weight of the gastrocnemius muscle and the area of muscle bundles were notably greater in the PCL-amnion group than in the chitosan group, and the sensory and motor capabilities of the rats were enhanced. In both studies, PCL-amnion appeared to be a promising biomaterial with greater efficacy than chitosan. Further research is required to explore its therapeutic effects on different nerve injuries and its potential clinical applications.

The application of amnion coated with halofuginone alone and in conjunction with chitosan was compared in a rat uterine horn injury model. Both treatments caused a decrease in the percentage of animals developing adhesions, together with a lower percentage of animals experiencing moderate to severe adhesions compared to untreated controls. The effectiveness of these two treatments is believed to stem from the physical barrier provided by the collagen substrate combined with collagen synthesis inhibition by halofuginone, added to the potential prevention of fibroblast and macrophage attachment by the chitosan gel. Amnion alone did not prevent adhesions more effectively than untreated controls [131].

The association of chitosan and fetal membranes was evaluated with a view to healing cutaneous burns, ocular surface lesions, and chronic wounds (Table 2).

Two studies evaluated the combined use of AM and chitosan in a rat cutaneous burn model. Momeni et al. investigated chitosan/PVP gel containing human AM extract for wound healing efficacy and scar preventive effects [135]. Epidermal and dermal regeneration were enhanced through the formation of granulation tissue, fibroblast proliferation, and blood capillary formation concomitant with collagen bundle emergence. Rana et al. evaluated the efficacy of hydrogels composed of amnion and collagen on cutaneous burn wound healing in rats [133]. Efficacy on re-epithelialization rate and time was compared using the gel in association with a covering membrane created from rabbit collagen and prawn shell chitosan (72 ± 3.27%, 16.75 ± 0.96 days) and without a covering membrane (62.5 ± 4.43%, 18.75 ± 0.50 days). Statistically significant results were obtained in favor of the combined use of hydrogel and chitosan-based covering membrane compared to the gel alone.

The pro-healing effect of AM extract combined with chitosan hydrogel and then seeded with AMSCs was tested in vitro using a scratch assay and gave promising results [137]. This method enhanced early angiogenesis, collagen deposition, wound closure, reepithelialization, and the formation of organized dermal epidermal junctions after 21 days of healing. These results suggest that hAMSC loaded on hydrogel composed of dAM extract and chitosan could offer a therapeutic strategy for the management of chronic wounds.

For ocular surface reconstruction, one disadvantage of AM graft was that bioactive factors were soon depleted after grafting. An improved AM grafting strategy has been tested using a nanoscale delivery system for the bioactive factors extracted from the human AM [134]. Nanoparticles prepared by polyelectrolyte complexation from chitosan and dextran sulfate to load AM extract prolonged and significantly increased biological activity in vitro. Nano-delivery of AM extracts appears to offer a promising option for optimizing the release of bioactive factors contained in AM. In vivo studies are needed to explore this potential.

An application of AM extract-loaded nanoparticles was also recently proposed to improve artificial corneas, a promising alternative for corneal transplantation. Haleh Bakhshandeh et al. demonstrated that the anti-angiogenic property of artificial corneas was significantly increased when decorated with AM extract-loaded nanoparticles [136]. Anti-angiogenic factors such as thrombospondin-1, endostatin, and heparin sulfate proteoglycan were released, and vascularization was inhibited through a decreased expression of cluster of differentiation 31 and von Willebrand factor.

Innovative dressings are still being developed to improve wound healing and tissue repair. Recently, Dadkhah Tehrani et al. [132] created a new wound dressing composed of an amniotic membrane with oxygen-generating capacity based on a chitosan thermosensitive hydrogel. This matrix was stable, released oxygen, had good mechanical properties, supported cellular growth, adhesion, and morphology, and did not cause any hemolysis or cytotoxicity as a result of H_2_O_2_ release. Given the importance of oxygen in wound healing, such a material could improve AM properties as a skin substitute. In vivo tests are now needed to develop these initial results.

### 4.2. Hyaluronic Acid

Hyaluronic acid (HA) is a natural glycosaminoglycan and a crucial element of the extracellular matrix. In fetal membranes, it is present in high concentrations in the amnion, and especially at the interface between the amnion and the chorion, also called the spongy layer [20].

HA presents diverse biological functions that make it useful for medical applications. It is biocompatible and able to promote wound healing by regulating inflammation, enhancing migration and proliferation of fibroblasts and keratinocytes, and stimulating the angiogenic ability of endothelial cells. HA has been extensively studied in the context of burns and chronic wounds. Its role is both structural and signaling when HA interacts with its binding molecules [139]. Limitations for clinical use include rapid degradation, weak mechanical properties, and poor adhesion [140]. The development of pharmaceutical forms of HA and derivatives has yielded products with increased residence time in human tissues and anti-inflammatory properties. Diverse forms of biomaterial are available, such as membranes, dressings, meshes, gels, and tubes. They find use as wound dressings, anti-adhesive agents, and scaffolds for tissue engineering applications including epidermal, dermal, microvascular skin, cartilage, and bone regeneration [141]. Hyaluronic acid is involved in numerous phases of the wound healing process. It performs these functions both structurally, by providing a suitable wound microenvironment for cell migration and through the regulation of various signaling pathways involved in tissue repair [142]. When hyaluronan interacts with its main transmembrane receptor, CD44, it can induce fibroblast migration and promote wound healing. This effect seems mediated by two tyrosine kinases: p185HER2 and c-Src [142,143,144]. Other signaling pathways can be activated by HA in the cells; they are mediated by Toll-like receptor (TLR), hyaluronan receptor for endocytosis (HARE), receptor for hyaluronan-mediated motility (RHAMM), and lymphatic vessel endothelial receptor 1 (LYVE-1). Upon interaction with HA, transmembrane receptors present on the cells induce intracellular signaling, allowing transcriptional regulation of cytokines, chemokines, and growth factors [145]. The anti-inflammatory property of high molecular weight HA (HMWHA) has been well documented in osteoarthritis. This function was mediated by CD44 and TLR receptors, leading to inhibition of the inflammatory cascade. The molecular mechanism still remains unclear, but it was hypothesized that HMWHA may mask TLR2 and TLR4 to inhibit their activation [146]. Low-molecular-weight HA (LMWHA) exhibits the opposite effect and is able to promote inflammation.

Hyaluronan also exhibits a dual effect on angiogenesis, depending on its molecular weight: LMWHA induces proliferation, migration, and tubule formation, whereas HMWHA is antiangiogenic. The molecular mechanism was not fully understood but seems to be mediated by CD44 and RHAMM, which are present on endothelial cells. Antimicrobial activity of HA has also been demonstrated. In intestinal epithelium, HA was able to enhance β-defensin 2, an antimicrobial peptide. This process was mediated by CD44 and TLR4 signaling pathways [147].

The involvement of HA in the healing process makes it a compound of choice for clinical applications. The use of HA in combination with fetal membranes could potentiate the anti-inflammatory and antimicrobial effects of the membranes through interaction with these receptors present on cells of the wound edges, activation or inhibition of corresponding signaling pathways resulting in transcriptional regulation of cytokines and growth factors. Moreover, the use of adhesive HA hydrogel could overcome the practical difficulties of using AM itself on skin and provide a microenvironment favoring re-epithelialization.

We searched literature on hyaluronan and fetal membranes in tissue repair and found no result involving chorion. Studies comparing HA and AM were presented in Table 3. Those evaluating the association of HA and AM for tissue repair were detailed in Table 3.

**Table 3:** Amniotic membranes and hyaluronic acid as promising strategies for tissue repair.

Reference	Material		Intended Clinical Application	Type of Study	Model	Evaluation Time	Biological Properties
A—Comparison of Hyaluronic Acid and Amniotic Membranes							
Szabo A. et al., 2000 [148]	AM	Serafim	Preventing adhesion after mesh repair of abdominal wall hernia	in vivo (animal model)	Rat (*n* = 60)	6 weeks	AM and Seprafilm were equally effective in preventing adhesions (area adhesion formation: AM: 0.96%, Seprafilm: 0%)
Fiorica C. et al., 2011 [82]	Collagen gel	HA/PHEA-EDA films	Coating for contact lenses (able to release limbal cells)	in vitro	HCEC, RLEC, RLF	14 days	Adhesion of primary rabbit limbal cells until 3 days, and then viable cells were released from the hydrogel surface
Hortensius R.A. et al., 2016 [149]	CG scaffolds with AM	CG scaffolds with HA	Tendon wound repair	in vitro	Horse tenocytes	7 days	Increased metabolic activity of tendon cells within AM scaffolds, AM, and HA tempered the expression of genes associated with the inflammatory response
B—Combined use of Hyaluronic Acid and Amniotic Membranes							
Ozgenel G.Y. et al., 2004 [150]	Repair site wrapped with AM and HA injected into it		Tendon wound repair	in vivo (*n* = 144 tendons)	Chicken	20 weeks	Effective in preventing adhesions of the flexor tendon
Zhang Y. et al., 2022 [140]	Adhesive hyaluronic acid hydrogel with AM-CM		Cutaneous wounds	in vitro and in vivo *(n* = not available)	HUVECs, db/db mice	12 days	Enhanced healing by regulating macrophage polarization and promoting angiogenesis
Corrêa MEAB, 2022 [151]	dAM solubilized with HA		Cutaneous wounds	in vivo (*n* = 96)	Rats	14 days	Reduced the acute inflammatory response with an earlier repair phase
Murphy S.V. et al., 2017 [8]	Solubilized AM-HA hydrogel		Cutaneous wounds	in vivo (n = not available)	Mice	14 days	Accelerated wound closure, re-epithelialization, increased total number of blood vessels, and proliferating keratinocytes within the epidermis
Mohammad J.A. et al., 2000 [152]	AM tube nerve conduit delivering NGF-HA		Nerve regeneration	in vivo (n = not available)	Rabbits	3 months	AM promoted biochemical factors, in combination with NGF/HA: enhanced nerve regeneration

HA: hyaluronic acid; Seprafine: membrane composed of carboxymethylcellulose and hyaluronic acid; HA/PHEA-EDA: hyaluronic acid chemically cross-linked with α,β-poly(N-2-hydroxyethyl) (2-aminoethylcarbamate)-d,l-aspartamide; HCEC: human corneal epithelial cells; RLEC: rabbit limbal epithelial cells; RLF: rabbit limbal fibroblasts; CG: collagen–glycosaminoglycan; AM-CM: lyophilized amnion-derived conditioned medium; HUVECs: human umbilical vein endothelial cells; dAM: decellularized amniotic membrane; NGF: nerve growth factors.

AM and Seprafilm composed of HA were evaluated after mesh surgery repair of abdominal wall hernia in a rat model. Both were equally effective in preventing adhesions after surgery [148].

In ophthalmology, AM substitutes are being developed to avoid the risk of infections and short-term degradation inherent to their use. The coating surface should be biocompatible and allow the attachment of limbal stem cells and their subsequent release for corneal regeneration [153]. Several synthetic materials have already been tested, including HA chemically cross-linked with α,β-poly(*N*-2-hydroxyethyl)(2-aminoethylcarbamate)-d,l-aspartamide. This material was able to attach to the cells and release viable cells after 3 days, suggesting a potential application for delivering limbal cells in the treatment of corneal damage [82]. This type of film was proposed as an AM substitute, but no study was found comparing it directly with AM. The positive control used in this study was a collagen gel, also investigated as an AM substitute in this context.

HA and AM have been investigated for tendon healing. After injury, disorganized collagen matrix appears and leads to scar formation, possibly due to the inflammatory phase of wound healing. Biomaterials used for tendon regeneration are expected to limit the inflammatory response. In this context, collagen–glycosaminoglycan (CG) scaffolds have been investigated in combination with HA or AM [149]. Scaffolds containing AM showed improved mechanical properties, and tendon cells within these scaffolds had increased metabolic activity. Both scaffolds (containing HA or AM) tempered the expression of genes associated with the inflammatory response.

Combined use of AM and HA has been tested with a view to healing cutaneous wounds, preventing adhesions after surgery for tendon repair, and enhancing axonal regeneration.

Adhesive HA hydrogel was tested as a carrier to deliver AM secretome in vitro and in vivo in one study. This experimental gel released AM factors gradually for at least 35 days without decline and increased healing of diabetic mouse wounds by regulating macrophage polarization and promoting angiogenesis [140].

Application of decellularized AM solubilized with HA on cutaneous wounds in rats showed an accelerated inflammatory process and an earlier repair phase [151]. Solubilized AM-HA hydrogel likewise yielded promising results on cutaneous wounds in a mouse model with accelerated wound closure, increasing total number of blood vessels, and proliferation of keratinocytes within the epidermis [8]. The same team compared this hydrogel to other amnion derivatives in a more recent study and confirmed these positive results of AM-HA hydrogel, with equivalent results found for AM powder [154]. Hydrogel and powder were more beneficial than unprocessed or cryopreserved AM sheets.

Use of AM and HA simultaneously was evaluated in tendon repair in 2004 and showed promising results on a chicken model for preventing adhesions after flexor tendon surgery. Fewest adhesions were observed when the repair site was wrapped with AM and HA was injected into it [150]. This association has never been used clinically.

One study found on the combined use of AM and HA for axonal regeneration. An experimental nerve conduit was produced from fresh AM and was used to deliver nerve growth factor (NGF) and HA continuously [152]. Axonal regeneration was measured in rabbits using this system to bridge a 25 mm nerve gap. Axonal regeneration was enhanced by the NGF/HA treatment compared to the AM tube nerve conduit used alone (45% more myelinated axons). In addition, the AM tube released biochemical factors contributing to the nerve regeneration process.

### 4.3. Platelet-Derived Growth Factors

Platelet-derived growth factors (PDGFs) were classified as a natural material for wound healing by Trinh et al. (2022) [11].

Their critical roles in development are well documented, but their normal physiological functions in adults remain unclear. Elevated PDGF activity has been associated with various diseases, prompting the development of drugs inhibiting PDGF or their receptors. Recombinant human PDGF-BB (becaplermin) is currently used in clinical practice for wound healing through its pro-angiogenesis properties in the treatment of chronic neuropathic lower-extremity diabetic ulcers [155]. It has also been used in dental fields, such as oral and maxillofacial surgery, periodontology, and dental implantology. This growth factor is able to regulate mesenchymal cell activity and is involved in angiogenesis, as well as the activation of neutrophils and macrophages—key cells for early-stage wound healing. Various studies have shown that PDGF promotes cell proliferation and chemotaxis in periodontal cells. While it inhibits collagen production and mineralization, it was demonstrated to accelerate maturation of collagen chains by increasing LOX activity and SPARC expression [156].

The pro-healing properties of PDGF do not include anti-inflammatory, analgesic, and antimicrobial actions that are provided by fetal membranes. Both appear to have complementary modes of action to promote wound healing, especially in dentistry.

The use of fetal membranes and PDGF was described in two published clinical studies. The first was an RCT comparing the two strategies to heal diabetic foot ulcers. The second was a case series proposing an association of PDGF and fetal membranes for a periodontic application.

**Table 4:** Clinical applications of PDFG and fetal membranes in wound healing.

Reference	Material		Clinical Application	Type of Study	Model	Evaluation Time	Clinical Results
Mohammadi Tofigh A and Tajik M, 2022 [157]	Dehydrated AM	PDGF gel	DFU	RCT	Clinical study (*n* = 243)	12 weeks	AM dressing: better healing rate (87.6%)
Rosen PS et al., 2015 [158]	Application of recombinant PDGF and allograft of mesenchymal cells covered by amnion–chorion barrier		Mandibular Class III/IV furcations	Case series	Clinical study (*n* = 5)	6–30 months	Complete closure in three patients; one patient had two furcation sites converted to Class I, and one patient was without improvement

AM: amniotic membrane; DFU: diabetic foot ulcer; RCT: randomized controlled trial; PDGF: platelet-derived growth factor.

The randomized controlled trial was conducted on 243 patients with diabetic foot ulcers (DFUs) classified Grades 1 and 2 on Wagner’s scale, without infection, and adequate tissue blood flow. The best DFU healing improvement was found with dehydrated AM compared to the two other strategies. Percentages of area reduction of the wound at 12 weeks were 50%, 61.7%, and 87.6%, respectively, in groups treated by surgical debridement, platelet-derived growth factor, and dehydrated AM. Significant differences were observed over the entire period [157].

A case series proposed a strategy associating fetal membranes and PDGF to save teeth in cases of severe furcation. Treatment consisted of root management with topical application of recombinant PDGF, allograft with mesenchymal stem cells in the furcation, and covering of the site with a fetal membrane-derived barrier [158]. Among the five included patients, three had complete closure, one had two furcation sites converted to Class I, and one patient showed no improvement.

### 4.4. Honey

Bee honey is a viscous, supersaturated solution composed mainly of fructose, glucose, water, and larger sugars. The remaining 1.5% comprises minerals, vitamins, amino acids, organic acids, flavonoids, and other phenolic compounds and aromatic substances.

Honey has been used in medicine for its many health benefits. Possessing anti-inflammatory, antioxidant, and antimicrobial effects, it was traditionally used on skin to treat burns, trauma, and chronic wounds until the advent of modern medicine [159]. The antimicrobial effect of honey has been attributed to the generation of hydrogen peroxide (H_2_O_2_) and to its osmotic properties, enabling it to absorb water from bacteria, thus preventing their growth [160]. The high osmolarity of honey also creates a moist environment conducive to wound healing and prevents it from adhering to new wound tissues. Additionally, one study demonstrated that honey stimulates the proliferation of fibroblasts and promotes angiogenesis [161]. The authors stated that “the influence of honey constituents on angiogenesis in a wound dressing context is likely to be positive but would depend on the effective dilution of the honey and the penetration of the active constituents against an osmotic gradient”. Tissue engineering approaches such as electrospinning and hydrogels are being developed to allow the active compound in honey to be delivered to the wound site and so enable its use in a range of other clinical areas, alone or in association with other materials [162].

A comparison of honey dressing and fetal membranes was found in two meta-analyses. One published study concerned an association of the two materials in a wound healing strategy (Table 5).

**Table 5:** Comparison and combined use of honey and amniotic membrane in wound healing.

Reference	Material		Clinical Application	Type of Study	Number of Patients	Evaluation Time	Clinical Results
Yang et al., 2021 [71]	AM	Other dressings: sulfadiazine, polyurethane membrane, and honey	Burn wounds	Meta-analysis (11 RCT)	*n* = 816	_	Honey > AM > conventional methods, silver sulfadiazine, and polyurethane membrane
Rahman et al., 2019 [163]	Biological dressing	Non-biological dressing	Skin graft donor site	Meta-analysis (8 RCT)	_	_	Biological dressing > non-biological dressing
Boyar and Galiczewski, 2018 [164]	Dehydrated AM allograft after autolytic debridement (active Leptospermum honey)		Deep neonatal wounds associated with extravasations	Case series	*n* = 3	2 months	AM: effective, safe, and easy-to-apply treatment leading to regeneration and healing

AM: amniotic membrane; RCT: randomized controlled trial.

The meta-analysis of Yang et al. [71] compared AM and other dressings for healing burn wounds. They included 11 RCTs representing 816 patients. Honey was found to be the best dressing before AM.

The one study associating honey and fetal membranes as a wound healing strategy was for the treatment of severe extravasation injuries in preterm neonates [164]. In this case series, three neonatal patients were first-line treated with autolytic debridement using honey, followed by mechanical debridement. Some days later (11–15 days) and with failure to close, a dehydrated AM allograft was placed and covered by surgical tape strips in two cases out of three. Closure was observed after one (two cases) or two (one case) allograft applications. Honey application continued until complete closure. Healing showed minimal contractures, soft scars, and normal pigmentation. Despite the frequency of these injuries in neonates, management intervention studies are of low quality, with no consensus in treatments or outcomes [165]. In this case series, the strategy of combining honey and AM allograft was effective, safe, and easy to apply.

Dehydrated AM contributed to promoting healing through extracellular matrix generation, which was faster compared to other treatments. The combined use of honey provides complementary benefits to fetal membranes, in particular by providing a moist environment favoring healing and probably the maintenance of factors secreted by the amnion.

### 4.5. Epigallocatechin-3-Gallate

Epigallocatechin-3-gallate (EGCG) is the major catechin in green tea [*Camellia sinensis* L. Ktze. (Theaceae)]. This polyphenol is believed to account for the health benefits ascribed to the consumption of green tea, which include antioxidant effects, cancer chemoprevention, improved cardiovascular health, enhanced weight loss, and protection of skin from damage caused by ionizing radiation [166].

EGCG has already been used in combination with decellularized AM in a diabetic context. Diabetic patients exhibit delayed wound healing, explained in part by impaired angiogenesis, an imbalance in the expression of cytokines and growth factors, increased metalloproteinase activity, and a chronic inflammatory state mediated by increased oxidative stress. Chronic inflammation is associated with persistent NLRP3 inflammasome activation in the wound, defects in macrophage polarization, and intensified neutrophil extracellular traps [167,168,169,170]. The roles of EGCG in promoting wound healing include limitation of neutrophil infiltration, inhibition of monocyte migration and adhesion, enhancement of re-epithelialization, reduction in ECM synthesis, and angiogenesis promotion [171]. Mechanisms involved in the wound healing process were described in numerous in vitro studies on cell lines and few studies on animal models. These studies suggest that EGCG enhances skin wound healing through its antioxidant, anti-inflammatory, antimicrobial, angiogenic, and antifibrotic properties by targeting both the NF-κB and the STAT3 signaling pathways. These properties make it a suitable candidate for promoting healing in diabetes.

The association of decellularized AM and EGCG was tested on a diabetic wound rat model and compared to rats treated with dAM or EGCG alone and to untreated controls [172]. The best results in terms of healing were obtained in the group combining dAM and EGCG, which synergistically decreased inflammation and increased cell proliferation, collagen deposition, and angiogenesis. As the beneficial properties of both compounds encompass all stages of the healing process, it is not surprising to obtain synergistic results when used in combination. Decellularization of the amnion allows to maintain the natural 3D structure and biochemical composition of AM and create a scaffold suitable to promote cell adherence and proliferation [173]. The cytokines and chemokines supplied by amnion could act in conjunction with EGCG to inhibit inflammation via different signaling pathways (via NF-κB and STAT3 for EGCG and via reduction in inflammatory characteristics of the immune cells by dAM). This while promoting re-epithelialization both through the extracellular matrix of amnion and through the signaling pathways activated by EGCG.

Given its antioxidant properties, EGCG was also tested in the preparation of dried AM in association with saccharide lyoprotectants with the aim of improving wound dressings used in ophthalmologic surgery [174].

### 4.6. Lysine

Lysine is an essential amino acid required to build proteins in the body. It has been studied for various applications, including for its ability to generate cross-links and so induce self-assembly of nanoparticles. Its molecular bridging effect was tested on carbodiimide cross-linked AM to create a more stable and biocompatible scaffold for corneal epithelial tissue engineering [175]. This study concluded that l-lysine pretreatment in the range 1–30 mM was a useful strategy to develop a stable limbal stem cell niche based on chemically cross-linked AM.

### 4.7. Aloe vera

*Aloe vera* is a succulent plant composed mainly of water (99–99.5%). Over 75 potentially active compounds have been identified in the remaining 0.5–1.0% of the plant, including vitamins, minerals, enzymes, polysaccharides, phenolic compounds, and organic acids [176].

*Aloe vera* has already been used for its pro-healing properties in the treatment of skin wounds and first-to-second-degree burns. Biological properties of *Aloe vera* related to wound healing include anti-inflammatory effects, increased collagen synthesis, and remodeling, thus promoting re-epithelialization. These properties were mainly attributed to polysaccharides, including mannose-6-phosphate. *Aloe vera* acts on immunomodulation through downregulation of inflammatory cytokines, NLRP3 inflamasome, cyclooxygenase pathway, PGE2 production, and decreased leukocyte aggregation [177,178]. Benefits of *Aloe vera* were also demonstrated in re-epithelialization via an increase in wound contraction and collagen synthesis. This effect was mediated by increased fibroblast proliferation and production of hyaluronic acid and hydroxyproline in fibroblasts.

In 2019, Rahman et al. tested a gel composed of AM and *Aloe vera* extract to heal skin burns in a rat model. They obtained promising results both in vitro and in vivo. The gel had the advantage of being easy to produce and to use on skin [163].

As both AM and *Aloe vera* exhibit anti-inflammatory properties and promote re-epithelialization, we can speculate that their combined use may lead to a synergic action. The amniotic membrane promotes focal adhesions and re-epithelialization through modulation of JNK, MEK MAP kinase, and TGF-ß signaling pathways, while *Aloe vera* also contributes to fibroblast proliferation and extracellular matrix remodeling. In addition, AM decreases inflammation in part through secretion of anti-inflammatory cytokines and suppression of pro-inflammatory cytokines, while *Aloe vera* also downregulates pro-inflammatory cytokines and modulates other signaling pathways leading to an anti-inflammatory environment. Molecular pathways of both materials remain not fully understood but seem to contribute together to the same anti-inflammatory and pro-healing effects.

## 5. Conclusions and Outlook

### 5.1. Main Content of the Article

Fetal membranes, historically used in ophthalmology and dermatology, are called upon to meet a growing demand for medical applications in a wide range of disciplines. Their use has been advocated in numerous applications, including surgical procedures, with safe and beneficial results. However, their clinical applicability remains narrow due to the lack of randomized controlled trials. The most advanced trials have been conducted on skin burns and diabetic foot ulcers.

In ophthalmology, amnion was previously used directly on the eye to repair the ocular surface. It is now considered a reference scaffold in stem cell therapy. Chitosan-based scaffolds have also been studied and have the advantage of improving biocompatibility and degradability compared to non-natural biomaterials [83]. The combined use of AM and chitosan as nanoparticles was tested in vitro and offered a promising option for optimizing the release of bioactive factors contained in AM at the ocular surface by prolonging their viability, thus increasing biological activity.

The use of AM on skin is mainly for burns and ulcers. In burn wounds, natural materials were suggested to provide better results than AM in two recent meta-analyses [71,74], especially honey and *Aloe vera*. However, the results of the second meta-analysis were published in Chinese, and we read only the abstract. The association of AM gel and *Aloe vera* extract to heal skin burns showed promising results in a rat model and would be easy to produce and to use on skin. This kind of material derived from AM could overcome the practical difficulties of using AM itself on skin. For the same purpose, adhesive hydrogels combining hyaluronic acid [8,139,149] or chitosan [135,137] with AM were developed and yielded good results in vitro and in animal studies. However, no studies comparing these compounds have yet been published.

Several RCTs have demonstrated the benefits and safety of using AM to promote healing of diabetic foot ulcers [56,57]. Dehydrated AM showed a better healing rate than PDGF gel in this context [157]. The cost of the three methods used in this RCT did not differ significantly.

AM wrapping was used in a pilot study for nerve repair [95] and was shown to be beneficial compared to chitosan hydrogel in an animal model [129,130].

The use of fetal membranes in dentistry was assessed in several pilot RCTs. Chorion gave promising results compared to amnion for treating periodontal intrabony defects and furcation defects. Application of recombinant PDGF associated with covering with an amnion–chorion barrier on severe furcation showed promising results in a clinical case series [158].

**Figure 5:**
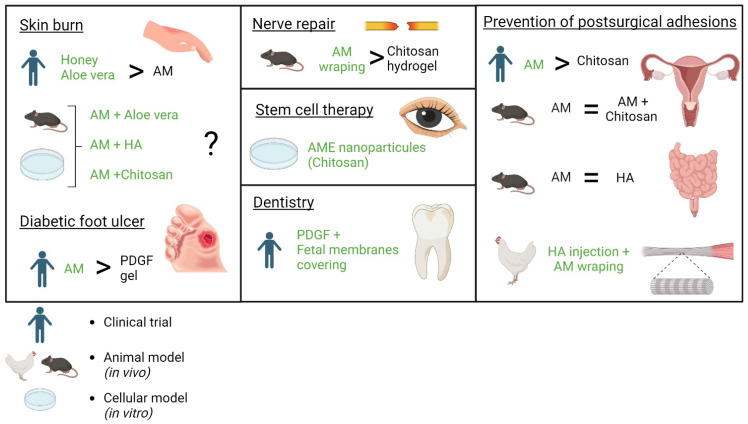
Fetal membranes and natural materials used for tissue repair. AM: amniotic membrane; HA: hyaluronic acid; PDGF: platelet-derived growth factor. Created in BioRender. Blanchon, L. (2024) BioRender.com/s92f110.

Amniotic membranes and natural materials have been reported to prevent postsurgical adhesions. In gynecology, fresh AM was beneficial compared to chitosan injection in preventing recurrence of IUA after transcervical resection of adhesion [124]. The application of amnion coated with halofuginone alone or in conjunction with chitosan in a rat uterine horn injury model showed an equivalent ability to reduce adhesion rate [131]. Hyaluronic acid and AM showed equivalent results for decreasing adhesions in a rat model of mesh repair of abdominal hernia [148]. Their use in association showed promising results in an animal model for tendon repair [150], but no further in vivo studies or clinical data have been published on this technique (Figure 5).

### 5.2. Challenges in the Field and Future Development Directions

Numerous commercial preparations derived from fetal membranes are available, as are dressings derived from natural pro-healing materials. The associated use of fetal membranes and natural materials has shown promising results, but further studies are needed to collect evidence for their efficacy and clinical relevance. The development of standardized research protocols and better comparison studies are needed to assess the true efficacy of these materials.

Recently, successful use of synthetic matrix systems for amniotic membranes has also been demonstrated in vitro and in vivo [179,180]. Gelatin methacrylate hydrogel appeared to be the most studied and was tested in association with several natural materials [181]. This raises questions about the use of natural matrices in comparison with synthetic ones. The main drawback of synthesized matrices is that they do not provide biological activity and necessitate supplementary substances in their composition to provide beneficial effect [179]. Biological matrices were proved to be beneficial compared to non-biological in a systematic meta-analysis including eight RCTs in the context of split-thickness skin-graft donor sites [52]. However, further reviews of the literature on other applications would be interesting. Natural matrices have the advantages of being highly biocompatible and bioactive and to degrade compatibly with the body’s natural processes [182].

Fetal membranes are a safe biological material with a wide range of clinical applications that continue to grow. However, despite many clinical studies, they have not been fully used for all suggested applications. Their use in clinical practice is challenging and highly dependent on preserving methods, which is essential to maintaining quality and safety. Cryopreservation being the most effective method for maintaining the biological activity of AM. However, it has drawbacks such as membrane shrinkage after thawing and the need for specialized equipment and facilities. Freeze-drying is more convenient and preserves the structural and biological properties of fetal membranes, but it can damage the tissue and is not ideal for long-term storage. Air-drying is the simplest and most cost-effective method, allowing the final product to be stored at room temperature, though it may result in the loss of biological activity and structural integrity [183]. Development of new products, such as a hydrogel delivery system for solubilized fetal membranes, could also be promising approaches to make clinical application easier [8,9].

## Supplementary Materials

The following supporting information can be downloaded at: https://www.mdpi.com/article/10.3390/ijms252211893/s1.

## Abbreviations

AM	amniotic membrane
AM-CM	lyophilized amnion-derived conditioned medium
AECs	amniotic epithelial cells
AMCs	amniotic mesenchymal cells
CG	collagen–glycosaminoglycan
DFU	diabetic foot ulcers
dAE	decellularized amnion extract
EGCG	epigallocatechin-3-gallate
ECM	extracellular matrix
HA	hyaluronic acid
HA/PHEA-EDA	hyaluronic acid chemically cross-linked with α,β-poly(N-2-hydroxyethyl) (2 aminoethylcarbamate)-d,l-aspartamide
HCEC	human corneal epithelial cells
HARE	hyaluronan receptor for endocytosis
HMWHA	high molecular weight HA
H_2_O_2_	hydrogen peroxide
HUVEC	human umbilical vein endothelial cells
IUA	intrauterine adhesion
LYVE-1	lymphatic vessel endothelial receptor 1
LMWHA	low-molecular-weight HA
MMP	matrix metalloproteinases
NGF	nerve growth factors
PCL	polycaprolactone
PDGF	platelet-derived growth factor
PLA	polylactic acid
PVA/NOCC	poly (vinyl alcohol)/N, O-carboxymethyl chitosan
RCT	randomized control trial
RHAMM	receptor for hyaluronan-mediated motility
RLEC	rabbit limbal epithelial cells
RLF	rabbit limbal fibroblasts
SOC	standard of care
TIMPs	tissue inhibitors of matrix metalloproteinases
TLR	Toll-like receptor
3T3 cells	mouse embryonic fibroblasts
