# Supplementary material for: Application of Fetal Membranes and Natural Materials for Wound and Tissue Repair
Source: Int J Mol Sci. 2024 Nov 5;25(22):11893. doi: 10.3390/ijms252211893 (PMC11594142; doi:10.3390/ijms252211893)
Supplement: Supplementary file 1 [file ijms-25-11893-s001.zip › ijms-3261769-supplementary.pdf]

Supplementary File S1

PubMed equation for articles on fetal membranes associated with or compared to natural materials

(extraction date : June 2024)

“("amnion"[MeSH Terms] OR "amnion"[Text Word] OR "amniotic"[Text Word] OR "amnions"[Text Word] OR ("chorion"[MeSH Terms] OR "chorion"[Text Word] OR "chorionic structure"[Text Word] OR "chorions"[Text Word] OR "chorionic"[Text Word]) OR ("extraembryonic membranes"[MeSH Terms] OR "extra-embryonic membranes"[Text Word] OR "membrane extraembryonic"[Text Word] OR "extra-embryonic membrane"[Text Word] OR "extra-embryonic membranes"[Text Word] OR "extraembryonic membrane"[Text Word] OR "membranes extra embryonic"[Text Word] OR "extraembryonic membranes"[Text Word] OR "Fetal membrane"[Text Word] OR "fetal membranes"[Text Word])) AND (“***material name***”[MeSH Terms] OR “***material name”***[Text Word])

Pro-healing natural materials whitout association with fetal membranes were :

4-Hydroxybenzaldehyde; 4-hydroxybenzoicacid ; 8-cineole ; A.asiaticaextract (AAE) ; acid folic ; Acteoside ; akuammidine ; alkaloids (echitamine) ; Allicin ; Aloe-emodin ; Alpha-mangostin ; amentoflavone ; anthocyanidines ; Arnebia nobilis ; Arnebin-1 ; Artocarpin ; ascorbic acid ; asiatic acid ; asiaticoside ; Astragaloside IV ; Astragalus membranaceus ; Azadirachta indica ; Bambusa bambos ; Beta-caryphyllene ; Beta-sitosterol ; Bexarotene ; Biophytum sensitivum ; Black seed ; Bletillastriata polysaccharide (BSP) ; Bryonia laciniosa ; C.officinalis extract ; C.officinalis hydroethanol extract (CEE) ; C.officinalistincture (CDOT) ; Cal. Officinalis ; Calendula officinalis ; calendula officinalis gel ; Calophyllolide ; camosol ; camphor ; caproic acid ; Carnosis acid ; Cassia auriculata ; Cassia roxburghii ; Catechin ; Cellulose gum ; Centella asiatica ; chlorogenic acid ; Chrysophanol ; Cinnamaldehyde ; cirsimaritin ; Citrus reticulate ; Coleus forskohlii ; crude extract of P.oleraceaL ; Cryptotanshinone ; Cucumin ; cyclicb-(1/3)(1/6)glucan/carrageenanhydrogels ; Dendrocalamus hamiltonii ; deoxyelephantopin ; dicaffeoylquinic acids ; diethylether extract (from M.sylvestrisandP.granatumflowers) ; dihydrokaempferol ; Dihydromyricetin ; Dihydroquercetin ; Drosera binata ; earthworm extract ; echitamidine ; ellagic acid ; Emodin ; Entadamide ; ethanolic ; Ferulic acid ; Ficus religiosa ; flavones ; flavonoids ; flavonols ; Fraxinus angustifolia ; gallic ; Gallic acid ; glycyrrhizin ; green tea ; Green-synthesized metal nanoparticles ; Guar gum ; Gum arabic ; Henna ; Hesperidin ; hexanic fractions ; horminone ; Isoacteoside ; Isoliquiritin ; Juglone ; kaempferol3-O-rutinoside ; Lansium domesticum ; lawsone ; Linseed ; Liquorice root extract ; Lupeol ; madecassic acid ; madecassoside ; malvaline ; malvidin ; malvin ; malvydyn ; malvyn ; Manuka honey (metylglyoxal) ; Methylglyoxal ; Momordica charantia ; Moringa oleifera ; Myricetin ; naphthaquinone ; naphthoquinones ; Naringenin ; Naringi crenulata ; naringin ; niacin ; Nyctanthes arbor-tristis L ; Orchidantha chinensis ; Panaxnotoginsengsaponins ; Physcion ; Phytophthora infestans ; Pinocembrin ; Pinocembrinanditslinolenoyl ester ; Piper nigrum ; Pluchea indica ; polysaccharidehydro gel ; polysaccharideIpomose ; polysaccharideβ-glucans ; Potato starch ; proazulenes ; protocatechuic acid ; quercetin-3-O-arabinosyl(1→6)glucoside ; Quinones ; Rhein ; rosmarinic ; rosmarinic acid ; S.officinalisleaf extract ; Saffron ; Saponin ; Saponins ; schaftoside ; Sesamol ; sesquiterpenelactones ; Silybum marianum L ; Snail extracts ; Spartium junceum L ; Squalene ; steroid alglyco alkaloid ; syringic acid ; Tannin ; Taspine ; Tecomella undulata ;Terpenoids ; Tocopherols ; Tragacanth gum ; trans-thujone ; tri terpenoid saponins ; Triterpenoids ; Turmeric ; Tyrosol ; Vanillin ; vitamin C ; vitamin D ; Vitamin E ; voacangine ; water fraction of hydroethanol extract ; WCEE ; Cellulose acetate
